# Detection of hepatitis C virus (HCV) RNA in the peripheral blood mononuclear cells of HCV-infected patients following sustained virologic response

**DOI:** 10.1007/s10238-022-00791-7

**Published:** 2022-01-23

**Authors:** Mahmoud A. Khattab, Yehia Zakaria, Eslam Sadek, Aliaa S. Abd EL Fatah, Magdy Fouad, Muhammed Khattab, Hend M. Moness, Nashwa Mohamed Adel, Elham Ahmed

**Affiliations:** 1 Present Address: Internal Medicine Department, Faculty of Medicine, Minia University, Cornish Al-Nile Road, PO: 61111, Minia, Egypt; 2 Tropical Medicine Department, Faculty of Medicine, Minia University, PO: 61111, Minia, Egypt; 3 Department of General Surgery, Hepatobiliary Unit, Faculty of Medicine, Minia University, PO: 61111, Minia, Egypt; 4 Clinical Pathology Department, Faculty of Medicine, Minia University, PO: 61111, Minia, Egypt; 5 Radio Diagnosis Department, Minia University, PO: 61111, Minia, Egypt

**Keywords:** Occult hepatitis C infection, Mononuclear cells

## Abstract

It is unclear whether direct-acting antiviral drugs (DAAs) result in the complete eradication of HCV infection or whether some quantities of the virus may persist after achieving a sustained virologic response (SVR). *Aim* The aim of this work was to study the possibility of the persistence of HCV RNA in peripheral blood mononuclear cells (PBMCs) after achieving SVR following DAA treatment. This study included 100 patients infected with HCV genotype 4, who were candidates for receiving DAAs and who achieved SVR during follow-up, as determined at 12 and/or 24 weeks following the end of treatment. All patients were subjected to demographic, biochemical and hematological assessments. Detection of HCV RNA in the serum and PBMCs and determination of the HCV genotype were performed with real-time PCR. We detected HCV RNA in the PBMCs of 20 out of 100 (20%) patients infected with HCV genotype 4, who achieved SVR. However, the persistent viral load in the PBMCs was very low (range: 400–900 U/mL; mean ± SD: 645.45 ± 153 U/mL). Multiple logistic regression analysis showed that only the higher posttreatment levels of aspartate transaminase (AST) were significantly predictive of HCV RNA persistence in the PBMCs (OR: 1.29; 95% CI: 1.08–1.55). Additionally, according to the Cox proportional hazard model, liver cirrhosis was the only significant risk factor for the persistence of HCV infection in PBMCs (HR: 5.8; 95% CI: 1.3–26.1; *P* < 0.02). Our results indicated the persistence of HCV RNA in some HCV patients who achieved SVR after treatment with DAAs.

## Introduction

Occult hepatitis C virus infection (OCI) is characterized by the presence of HCV RNA in the liver cells and/or the peripheral blood mononuclear cells (PBMCs) of patients whose serum samples test negative for HCV RNA [1]. In recent years, the treatment options for hepatitis C virus infection have been revolutionized by the development and use of IFN-free oral direct-acting antiviral agents (DAAs) for the treatment of HCV infection. These DAAs, which target the nonstructural viral proteins, have achieved eradication of the virus at a rate approaching 100% regardless of liver fibrosis, prior response to IFN/RBV, sex, age and race [2]. However, it is unclear whether the current antiviral treatment for chronic hepatitis C virus (HCV) infection with direct-acting antiviral agents (DAAs) results in the complete elimination of the virus or whether small quantities of the virus may persist. Although HCV is a hepatotropic virus, there is ample evidence to suggest that it can also replicate in peripheral blood mononuclear cells (PBMCs), and the infected cells have been reported to contain the negative strand of the HCV RNA, which is the viral replicative intermediate [3]. It has been suggested that HCV, by infecting the PBMCs, can use them as a reservoir that may contribute to the development of resistance to treatment, relapse and the possible occurrence of complications even after clearing of the HCV RNA from the serum of infected patients [4]. On the other hand, HCV-RNA persistence in PBMC in certain patients who achieved HCV RNA clearance is associated with persistent histological abnormality [5]. SVR after DAA treatment may need dual testing in both serum and PBMCs^.^[6].

This study aimed to investigate the possible existence of OCI following SVR after DAA treatment.

## Patients and methods

In this prospective cohort study, the recruited chronically HCV-infected patients attended our Minia University Liver Center and were candidates for receiving DAA treatment from 01/01/2017 to 31/9/2018. The eligibility criteria for receiving DAA therapy were consistent with the protocol approved by the national committee for the control of viral hepatitis in Egypt (NCCVH)_._[7] The patients with negative serum HCV-RNA as tested by PCR at 12 and/or 24 weeks after the end of antiviral treatment were diagnosed with sustained virologic response (SVR). Only patients infected with HCV genotype 4 who achieved SVR were included in this study. Selection of the study patients was performed regardless of the state of the liver parenchyma (cirrhotic or not) and previous treatment (experienced or naive patients). The exclusion criteria included coinfection with hepatitis B virus (HBV) or human immunodeficiency virus (HIV), patients with liver malignancies or with suspicious nodules on radiologic examination, previous or current use of immunosuppressive drugs and chronic liver diseases other than HCV infection. All patients signed informed written consent forms before participating in the study. The study protocol was approved by the Institutional Research Board of the Minia Faculty of Medicine in Egypt. The study was conducted in accordance with the ethical guidelines in the 1975 Helsinki declaration.

### Methods

In the recruited patients, the diagnosis of cirrhosis was made according to the clinical and sonographic criteria for cirrhosis and was confirmed by FibroScan [8, 9]. The patients were supplied with one of the available treatment regimens, predominantly sofosbuvir/daclatasvir/ribavirin. Consistent with the NCCVH Egyptian protocol, the treatment duration was either 12 or 24 weeks according to the patient’s condition, whether treatment naïve, treatment experienced or cirrhotics. DAC/SOF, SIM/SOF or LED/SOF were used in treatment naïve and treatment experienced for 12 Wk. and DAC/SOF/RBV for 12 Wk or DAC/SOF for 24Wk were used in cirrhotics. Demographic data and a history of previous treatment failure with PEGylated interferon or DAAs were recorded. Additionally, a history of any concomitant chronic illness, such as diabetes mellitus and hypertension, was noted. A clinical examination was performed that included measurements of body weight and height, which were used to calculate the body mass index [BMI = Weight (kg)/Height2 (m2)] [10]. Additionally, the waist and hip circumferences were measured and used to calculate the waist-to-hip ratio (WHR) [11]. Laboratory investigations were performed in three stages. First, genotype 4 analysis was performed by real-time PCR using the TaqMan Gene Expression Assays Kit supplied by QIAamp Viral RNA Kit (Qiagen). Second, a complete blood count was performed with an automated cell counter (Sysmex KX-21 N (TAO Medical Incorporation, Japan), and liver and renal function tests were performed with the autoanalyzer Konelab i60 (Thermoelectro, Clinical chemistry automation systems, Finland). The INR was measured by a STAGO COMPACT CT coagulation analyzer (Diamond Diagnostics, USA), and HCV-RNA was detected in the serum by real-time PCR. The viral markers HBsAg and HIV Abs were tested with fully automated ChemiLuminescence technology (Cobas E 411-Roche-Roche Diagnostics GmbH Germany). All of these investigations were repeated 3–6 months after the end of treatment. We detected HCV-RNA in the serum by PCR 3 and/or 6 months after the end of antiviral treatment when the patient got the sustained virological response (SVR).B lood samples (5–10 mL) were withdrawn in HCV RG RT-PCR Kit tubes (red cap). Whole blood was separated into plasma and cellular components this was done by centrifuging whole blood for 20 min at 800–1600 g. 200 µL of whole blood cells were transferred to a sterile polypropylene tube for PBMC studies. Then plasma was transferred into another sterile polypropylene tube.

RNA isolation for plasma done by the QIAamp DSP Virus Kit (Cat. No. 61704; Qiagen).RNA was incubated with 1 pmol of an HCV 5NC primer RC21 for 8 min at 70 °C and 5 min at 4 °C. RNA template was then reverse transcribed at 60 °C for 1 h with7.5 U of ThermoscriptTM Reverse Transcriptase, 20 U of RNase Out, 10 mM DTT, 1 mM deoxyribonucleotide and cDNA buffer (50 mM Tris–acetate pH 8.4, 75 mM potassium acetate, 8 mM magnesium acetate) (GibcoBRL Life Technologies) in a final volume of 10 mL. A denaturation step was performed at 95 °C for 5 min and was followed by a RNaseH treatment with 1 U of E. coli RNaseH (GibcoBRL Life Technologies) at 37 °C for 20 min. The primers RC1 (5′-GTCTAGCCATGGCGTTAGTA-3′) and RC21 (5′-CTCCCGGGGCACTCGCAAGC-3′) were designed to amplify a 220 bp fragment within the 5′ non-coding region of the HCV genome.

Blood samples were drawn to test for HCV RNA in PBMCs. The detection was performed in two steps: 1) extraction of HCV-RNA from PBMCs and 2) detection and amplification of HCV by real-time PCR. In the extraction step, PBMCs were isolated from the peripheral blood of HCV-infected patients by density gradient centrifugation. TRIzol reagent was used to extract the HCV-RNA from the PBMCs. The TRIzol reagent is a ready-to-use reagent designed to isolate high-quality total RNA from cell and tissue samples from humans within one hour. TRIzol reagent is a monophasic solution of phenol, guanidine isothiocyanate and other proprietary components that facilitate the isolation of a variety of large or small RNA species (TRIZOL TM reagent cat. No. 15596018 supplied by SIGMA) [12]. The extracted samples were then stored in a − 80 °C refrigerator prior to the next step. Detection of HCV RNA strands in PBMCs was carried out as described by Lohr et al. [13]. Detection and amplification of HCV were performed using DT-Lite 48 (DNA Technology, Russia). We added 10 μl from the extracted sample. The standard control (from 1–4) was added (10 μl) to separate tubes. Then, each tube was capped and placed in the real-time PCR instrument. We had four standard controls, and their concentrations were as follows: Concentration (1) was (1 ~ 5 × 10 7 IU/mL), Concentration (2) was (1 ~ 5 × 10 6 IU/mL), Concentration (3) was (1 ~ 5 × 10 5 IU/mL) and Concentration (4) was (1 ~ 5 × 10 4 IU/mL).

The lower detection limit was 10 IU/ml. According to the positivity or negativity for HCV RNA in the PBMCs after achieving SVR, the studied patients were classified into two groups.

### Biostatistical analysis

The statistical methods of this study were reviewed by Dr. Amani Mohammed, Minia University. Data are reported as medians and ranges, unless stated otherwise. Statistical analyses were performed with IBM SPSS software version 23 (IBM, New York, USA). The Kolmogorov–Smirnov test for normality was used to differentiate between parametric and nonparametric data. For the analysis of quantitative data, independent sample t tests were used, as appropriate. The chi-square (X^2^) test and Fisher’s exact test were applied to analyze qualitative data. Regression analysis was performed to detect the variables that were significantly associated with the persistence of HCV RNA in PBMCs after SVR. Cox hazard regression analysis was carried out to determine the risk factors associated with the persistence of the virus in PBMCs after achieving SVR. For all tests, the probability (p) was considered nonsignificant if ≥ 0.05 and significant if < 0.05.

## Results

In total, 100 patients with chronic hepatitis C infection and SVR at 3/6 months were included in this study. HCV RNA was detected in the PBMCs of 20 out of the 100 (20%) patients with SVR who participated in the study.

Table 1 presents the demographic and descriptive analysis of the study data. The study patients included 53 (53%) males and 47 (47%) females, with a mean age of 57.5 years (range: 23–75 years). The mean BMI was 29.4 kg/m^2^, with a range from 18.5 to 48.3 kg/m^2^_._ Our data showed that 35% of our subjects were hypertensive, and 29% were diabetic. The percentage of noncirrhotic patients was 51% versus 49% who had liver cirrhosis; those with liver cirrhosis were further classified according to the Child-Turcotte-Pugh (CTP) classification system, with 81.6% classified as CTP class (A), 16.3% as CTP class (B) and 2% as CTP class (C). The distribution of treatment regimens was as follows: sofosbuvir/daclatasvir/ribavirin regimen (39%), sofosbuvir/daclatasvir (20%), sofosbuvir/ribavirin (20%) and others (21%). Additionally, our data showed that 73% of subjects received a 12-week regimen, while only 27% received treatment for 24 weeks. Among the 100 patients who received the antiviral treatment, 90 (90%) were naïve to treatment, while only 10 patients (10%) had been treated before by interferon/ribavirin and this time we used DAC/SOF, SIM/SOF or LED/SOF for 12 Wk.

**Table 1:** Sociodemographic descriptive statistics for the study populations (100 patients)

	Mean ± SD or *N* (%) (range)
Age (years)	57.5 ± 11.2
	(23–75)
BMI* (kg/m^2^)	29.4 ± 5.4
	(18.5–48.3)
WHR*	0.9 ± 0.07
	(0.8–1.13)
*Sex*	
Male	53 (53%)
Female	47 (47%)
*Residence*	
Urban	32 (32%)
Rural	68 (68%)
*Smoking*	
Yes	18 (18%)
No	76 (76%)
Ex-smoker	6 (6%)
*HTN*	
Yes	35 (35%)
No	65 (65%)
*DM*	
Yes	29 (29%)
No	71 (71%)
*History of hematemesis*	
Yes	3 (3%)
No	97 (97%)
*Lower limb edema*	
Yes	22 (22%)
No	78 (78%)
*Cirrhosis*	
Noncirrhotic	51 (51%)
Cirrhotic	49 (49%)
*Child–Pugh class (among cirrhotic patients)*	
A	40 (81.6%)
B	8 (16.3%)
C	1 (2%)
*Type of treatment*	
SOF and DAC	20 (20%)
SOF, IFN and RIB	12 (12%)
SOF and LED	1 (1%)
SOF and IFN	3 (3%)
SOF and RIB	20 (20%)
SOF, DAC and RIB	39 (39%)
SOF and SIM	2 (2%)
SOF, LED and RIB	2 (2%)
OMB, PAR and RIT	1 (1%)
*Previous treatment failure*	
Yes	10 (10%)
No	90 (90%)
*Duration of treatment*	
3 months	73 (73%)
6 months	27 (27%)

*BMI* Body mass index, *WHR* Waist/hip ratio

Regarding the impact of treatment regimens on the persistence of HCV, there was no significant relationship between the treatment type (regimen) and the positivity for viral RNA in the PBMCs (*P* = 0.7). Additionally, there was no difference in PBMC HCV RNA positivity between patients who were treated for 12 or 24 weeks (*P* = 0.3). Both the negative and positive PBMC HCV RNA groups had the same percentages of subjects who had been treated previously (10%); *P* = 1. At the same time, the pretreatment viral loads were not significantly different between the HCV RNA positive and negative groups (Table 2). We found a significantly lower posttreatment prothrombin concentration (PC %) in the HCV RNA-positive group than in the negative group (Mean ± SD = 84.9 ± 16.2 vs. 93 ± 11.2, respectively, with p value = 0.01). Similarly, the International Normalized Ratio (INR) was significantly higher in the positive group than in the negative group (mean ± SD = 1.09 ± 0.14 vs. 1.03 ± 0.07, respectively, with *P* value = 0.01). Furthermore, both the posttreatment ALT and AST levels were significantly higher in the HCV RNA-positive group than in the negative group (*P* < 0.0001 for both) (Table 3). The posttreatment CBC and other biochemical profiles were not significantly different between the groups. In our study, we followed 20 patients with PBMCs that tested positive for the presence of HCV RNA 24 weeks after treatment; PCR was performed with serum samples obtained from these patients, and it was revealed that only one out of the 20 patients developed overt HCV relapse (Table 4).

**Table 2:** Bivariate statistical analysis for pretreatment variables in patients with PBMCs positive and negative for HCV RNA

	Group 1 − ve PCR in PBMCs *N* = 80	Group 2 + ve PCR in PBMCs *N* = 20	*P* value
*Age (years)*			
Range	23–75	32–72	0.8
Mean ± SD	57.3 ± 11.4	58 ± 10.5	
*Duration of treatment*			
3 months	60 (75%)	13 (65%)	0.3
6 months	20 (25%)	7 (35%)	
Previous treatment failure			
Yes	8 (10%)	2(10%)	1.00
No	72(90%)	18(90%)	
*Type of treatment*			
SOF and DAC	17 (21.2%)	3 (15%)	0.7
SOF, IFN and RIB	10 (12.5%)	2 (10%)	
SOF and LED	1 (1.2%)	0 (0%)	
SOF and IFN	3 (3.8%)	0 (0%)	
SOF and RIB	13 (16.2%)	7 (35%)	
SOF, DAC and RIB	31 (38.8%)	8 (40%)	
SOF and SIM	2 (2.5%)	0 (0%)	
SOF, LED and RIB	2 (2.5%)	0 (0%)	
OMB, PAR and RIT	1 (1.2%)	0 (0%)	
Hb (g/dl)			
Range	8–16	9.5–15.5	0.5
Mean ± SD	12.4 ± 1.6	12.1 ± 1.7	
*TLC (*× *10*^*9*^*/L)*			
Range	2500–10,400	2800–7600	0.04*
Mean ± SD	5836.2 ± 1996.3	5040 ± 1425.8	
*PCR (IU/mL)*			
Range	1534–13,646,369	14,498–7,301,515	0.4
Mean ± SD	962,010 ± 1,912,916	1,306,913 ± 2,084,741	
*Platelet count (10*^*9*^*/L)*			
Range	44–432	49–413	0.43
Median	170.5	152	
Mean ± SD	188.7 ± 87.5	174.2 ± 95.1	
*Creatinine (mg/dL)*			
Range	0.5–1.9	0.6–1.8	0.9
Mean ± SD	0.88 ± 0.2	0.89 ± 0.3	
*Albumin (g/dL)*			
Range	3–5	3.1–4.5	0.4
Mean ± SD	4.1 ± 0.4	4 ± 0.4	
*Total bilirubin (mg/dL)*			
Range	0.3–3.2	0.3–2.4	0.5
Mean ± SD	0.9 ± 0.5	0.8 ± 0.5	
ALT (U/L)			
Range	14–122	25–110	0.002*
Mean ± SD	32.1 ± 18.4	46.6 ± 18.7	
AST (U/L)			
Range	20–88	24–89	0.06
Mean ± SD	38.4 ± 13.8	45.3 ± 18.3

**Table 3:** Bivariate statistical analysis for posttreatment variables in patients with PBMCs positive and negative for HCV RNA

	Group 1 − ve PCR in PBMC *N* = 80	Group 2 + ve PCR in PBMC *N* = 20	*P* value
*Posttreatment PBMC PCR (IU/mL)*			
Range	Negative	400–900	< 0.001*
Mean ± SD		645.45 ± 153.2	
*Hb (g/dl)*			
Range	9.7–17.5	6.5–14.9	0.2
Mean ± SD	12.9 ± 1.4	12.5 ± 1.8	
Platelet count (× 10^9^/L)			
Range	45–422	65–415	0.7
Mean ± SD	202 ± 97.7	193.9 ± 110.8	
*TLC (*× *10*^*9*^*/L)*			
Range	2.8–11,100	2800–9600	0.05
Mean ± SD	5840 ± 2030	4865 ± 1781	
*PC%*			
Range	56–100	45.6–100	0.01*
Mean ± SD	93 ± 11.2	84.9 ± 16.2	
*INR*			
Range	1–1.32	1–1.51	0.01*
Mean ± SD	1.03 ± 0.07	1.09 ± 0.14	
*BMI (kg/m*^*2*^*)*			
Range	18.5–42.1	20.9–48.3	0.9
Mean ± SD	29.4 ± 5	29.3 ± 6.5	
*Waist to hip ratio*			
Range	0.85–1.13	0.8–1.11	0.4
Mean ± SD	0.9 ± 0.06	0.9 ± 0.08	
*ALT (U/L)*			
Range	11–80	19–330	0.0001*
Mean ± SD	26.1 ± 12.2	86.9 ± 75.8	
*AST (U/L)*			
Range	15–83	39–342	0.0001*
Mean ± SD	34.1 ± 13.3	96.8 ± 73.6	

*P* value calculated by chi-square test and independent sample *t*-test

**Table 4:** Follow up of patients with PBMCs positive for HCV RNA

PBMCs positive for HCV RNA N: 20 patients	
HCV RNA positive in serum	HCV RNA negative in serum
N:1patient (%5)	N:19 patients (%95)

Table 5 demonstrates the correlation between the posttreatment variables and the persistence of HCVRNA in the PBMCs; there is a highly significant positive correlation between the posttreatment levels of the liver enzymes ALT and AST and PBMC HCV RNA positivity (ALT had *r* = 0.57 and *P* < 0.0001, while AST had *r* = 0.61 and *P* < 0.0001). At the same time, both the posttreatment prothrombin concentration and the INR were correlated with PBMC HCV RNA positivity (*r* = − 0.28 and *P* = 0.004 for PC% and *r* = 0.26 and *P* = 0.008 for INR). Utilizing multiple regression analysis to identify the variables associated with the persistence of the HCV RNA in the PBMCs among the studied subjects, we observed that only the posttreatment liver enzyme AST had a significant predictive ability for the persistence of HCV RNA in the PBMCs (OR: 1.29; 95% CI: 1.08–1.55 and *P* = 0.005; Table 6). Using the Cox proportional hazard model to evaluate the effect of various variables on the persistence of HCV RNA in the PBMCs, we found that only the presence of liver cirrhosis was associated with a significant risk for PBMC HCV RNA positivity (HR: 5.828, 95% CI: 1.3–26.1 with *P* = 0.02). Other factors, such as age, sex and platelet count, liver enzyme levels, treatment type and treatment experience, had no significant effects (Table 7).

**Table 5:** Correlations between posttreatment investigations and positive HCV RNA in PBMCs among the studied subjects

	POSIIVE PCR in PBMC Spearman’s rho	
	*r*	*P* value
Posttreatment platelet count (× 10^9^/L)	− 0.04	0.6
Posttreatment HB (g/dl)	− 0.03	0.7
Posttreatment TLC (× 10^9^/L)	− 0.13	0.1
Posttreatment PC%	− 0.28	0.004*
Posttreatment INR	0.26	0.008*
Posttreatment ALT (U/L)	0.57	0.0001*
Posttreatment AST (U/L)	0.61	0.0001*

**Table 6:** Multiple regression analysis for posttreatment variables affecting the persistence of HCV RNA in PBMCs among the studied subjects

Independent variables	OR (95% CI)	*P* value
Age (years)	1.057 (0.91–1.22)	0.4
BMI (kg/m^2^)	0.96 (0.79–1.18)	0.7
Previous treatment failure	4.17 (0.08–200.3)	0.4
Cirrhosis	3.72 (0.21–66.2)	0.3
Treatment duration (months)	1.73 (0.7–4.2)	0.2
Hemoglobin (g/dl)	0.67 (0.36–1.25)	0.2
Platelet count (× 10^9^/L)	1.006 (0.99–1.02)	0.3
PC%	0.86 (0.62–1.19)	0.3
Posttreatment ALT (U/L)	0.89 (0.78–1.01)	0.08
Posttreatment AST (U/L)	1.29 (1.08–1.55)	0.005*

**Table 7:** Cox regression analysis for patient pretreatment variables and the persistence of HCV RNA in PBMCs

	HR (95% CI)	*P* value
Age (years)	1.026 (0.97–1.077)	0.2
Sex	2.588 (0.63–10.51)	0.1
BMI (kg/m^2^)	1.023 (0.93–1.121)	0.6
Cirrhosis	5.828 (1.30–26.1)	0.02*
Previous treatment failure	0.683 (0.13–3.351)	0.6
Pretreatment Hb (g/dl)	0.9 (0.7–1.2)	0.7
Pretreatment TLC (× 10^9^/L)	1 (1–1)	0.1
Pretreatment platelets (× 10^9^/L)	0.9 (0.99–1.006)	0.7
Pretreatment creatinine (mg/dL)	1.8 (0.3–10.05)	0.4
Pretreatment albumin (g/dL)	0.6 (0.2–2.03)	0.4
Pretreatment TB (mg/dL)	0.3 (0.1–1.3)	0.1
Pretreatment ALT (U/L)	1.02 (0.9–1.07)	0.1
Pretreatment AST (U/L)	0.99 (0.93–1.05)	0.7
Pretreatment PCR (IU/mL)	1 (1–1)	0.1

## Discussion

Our study has shown the existence of OCI among some HCV-infected patients who were treated with the recently available DAAs. Our data indicated persistence of HCV RNA in the PBMCs in 20 out of 100 (20%) patients infected with HCV genotype 4, who achieved SVR after 3/6 months of DAA treatment.

OCI was described by Pham et al. [14] as the presence of HCV RNA in the liver tissue and/or peripheral blood mononuclear cells (PBMC) in patients who tested negative for serum HCV RNA. At a later date, Pham et al. reported that the immune system, supports HCV replication regardless of the clinical appearance of infection and although HCV RNA occurs at a comparable frequency in all cell subtypes in CHC, monocytes contain the greatest loads. In contrast, B cells tended to carry the highest virus quantities during occult infection and monocytes appeared to be the least frequently infected [15].

Since its discovery, OCI has been documented in different populations, such as patients with cryptogenic hepatitis, hemodialysis patients, patients with cryoglobulinemia, HCC patients with undetermined etiology and even asymptomatic apparently healthy people [16, 17]. Additionally, there is an increasing amount of data suggesting that HCV relapses may represent activation of an occult hepatitis C virus infection [18]. Indeed, the gold standard for OCI diagnosis is detection of HCV RNA by nucleic acid amplification testing (NAT) from hepatocytes by liver biopsy. However, as liver biopsies are invasive, fairly high threat and are not readily obtainable, alternative diagnostic methods for detecting OCI developed [19]. The HCV-RNA detection rates in liver biopsy specimens varied widely among studies, reach to 83% [20, 21]. The detection rates of HCV-RNA in PBMCs have varied widely among studies investigating OCI, ranging from 0 to 50% [22–25]. For example, using a less sensitive assay to detect HCV-RNA, Carreno et al. found that 6 out of 12 (50%) individuals had detectable genomic HCV-RNA in PBMCs; the samples were obtained from anti-HCV antibody-positive patients who had normal ALT levels and whose serum had tested negative for HCV RNA for at least 12 months [20].

In contrast, Bernardin et al. [22] using a highly sensitive transcription-mediated amplification (TMA) test performed in duplicate assays, found that none of their 69 aviremic individuals with previous hepatitis C infections had detectable PBMC-associated HCV-RNA. At the opposite Sepideh Nasimzadeh et al., investigate the presence of occult HCV in beta-thalassemia patients, the plasma and PBMCs were collected from 90 beta-thalassemia patients. They used gene sequence which is a sensitive and accurate method than transcription-mediated amplification (TMA) test to detect presence of occult HCV in plasma and PBMCs. They found that 6.7% patients had OCI [26]. Mekky et al. is a multi-center study that was done in upper Egypt on 1280 genotype-4 HCV-infected patients who received SOF (400 mg) plus DCV (60 mg) once daily ± ribavirin regimen for 12 weeks and achieved SVR 12 weeks posttreatment found that HCV-RNA was detected in PBMCs of 50 (3.9%) of their patients. Detection of HCV viral load in the plasma and PBMCs was performed by standardized quantitative real-time PCR [26]. This study agreed with our study and they used the same method for detection of HCV in the serum and PBMC. The difference in results among Bernardin et al., Mekky et al. and our study may be due to difference in genotype.

Despite the studies providing evidence for the presence of OCI, there continues to be a controversy, with some authors challenging the existence of OCI [22, 27–29].

The finding of HCV RNA negative strands in the PBMCs in our OCI patients may indicate that those patients are candidates for future complications, and this finding agreed with the results of the study by Pawełczyk et al., in which it was reported that the clinical consequences of OCI may have a wide spectrum of presentations. OCI may be responsible for relapses, as it has been suggested that the clearance of identifiable HCV from the liver tissue and/or PBMCs according to highly sensitive reverse transcription and nested polymerase chain reaction assays may be a better indicator of a long-term sustained response than the absence of HCV-RNA in the serum or plasma [26]. In our opinion the problem is that the techniques of extraction of HCV-RNA from the PBMCs don’t present in all laboratories so, it will not be easily for HCV patients after treatment to be followed by these methods to detect the SVR. Additionally, diseases that affect PBMCs counts may give false negative results.

In our study, we followed 20 patients with PBMCs that tested positive for the presence of HCV RNA 24 weeks after treatment; PCR was performed with serum samples obtained from these patients, and it was revealed that only one out of the 20 patients developed overt HCV relapse.

In the new era of direct-acting antiviral agents, our study is the latest in a series of investigations suggesting the possibility of the persistence of HCV as an occult infection after achieving a putative SVR; our study focused on HCV genotype 4 in Egypt. The findings of this and other similar studies may raise questions about the current definitions of a sustained virologic response and the debate about the efficacy of the current short treatment durations.

This study investigated various variables that may impact the persistence of HCV RNA in PBMCs, correlating these factors and PBMC HCV RNA positivity. We found a highly significant positive correlation between high posttreatment levels of the liver enzymes ALT and AST and PBMC HCV RNA positivity (*P* < 0.0001). Additionally, there was a significant positive correlation between PBMC HCV RNA positivity and both a low prothrombin concentration and an elevated INR (*P* < 0.004 and *P* < 0.008, respectively). However, after multiple regression analyses, only the AST level was found to be significantly predictive of PBMC HCV RNA positivity (OR: 1.29; 95% CI: 1.08–1, 55; *P* < 0.005).

This finding may promote the hypothesis of OCI-related cryptogenic hepatitis, in which elevated liver enzymes may be a consequence of the persistence of HCV in a low replicative state in liver cells, lymphoid tissue (PBMC) or both. This is in agreement with the findings of Castillo et al. who described the presence of HCV RNA in liver tissue samples from 57 out of 100 patients who were negative for HCV antibodies and HCV RNA but had elevated liver enzyme levels [30].

Additionally, in a recent interesting study by Elmasry et al. [31] 134 patients with recurrent HCV infection following liver transplantation were recruited and treated with DAAs from 2014 through 2016. Out of the 129 patients who achieved SVR, the authors found that in more than 10% (no = 14) of the patients, the serum levels of aminotransferases did not normalize during or after DAA therapy. Of those 14 patients, nine were assessed for occult HCV infection by reverse transcription quantitative PCR of samples obtained from both liver tissue and PBMCs. Their results revealed that 55% of those nine patients (*n* = 5) had occult infections (three in liver tissue only, one in PBMCs only and one in both liver tissue and PBMCs), with the detection of the negative strand of the viral genome, indicating viral replication. Their findings are in agreement with ours, pointing to the presence of an underlying occult HCV infection in some patients as a possible cause for abnormal levels of serum aminotransferases in spite of achieving SVR 12 weeks after the end of DAA treatment.

However, an OCI can be present in the absence of elevated liver enzyme levels, as evidenced by Pham et al. [14], who reported OCIs in anti-HCV-positive patients who recovered after a self-limited (untreated) episode of hepatitis C and achieved SVR after interferon (IFN)-based treatment with normal liver enzyme levels.

Using the Cox proportional hazard model, we found a significant relationship between the presence of liver cirrhosis and the risk of developing an OCI (HR; 5.828; 95% CI; 1.3–26.1; *P* < 0.02). Similar data were reported by Rahman et al. [32], who concluded that late relapses can occur during long-term follow-up after SVR following IFN-based therapy. Importantly, they found that this occurred more often in patients with cirrhosis.

Similarly, Sood et al. [33] reported approximately 100 patients who achieved SVR and were followed for durations ranging from 6 months to 8 years. Eight of those 100 patients (8%) developed late recurrence. Late relapses were more frequent in patients with cirrhosis (5/28 [18%]) than in those without cirrhosis (3/72 [4%]; *P* = 0.037).

The pretreatment viral load, the treatment type and the treatment duration were not associated with the persistence of the virus in the PBMCs.

Although the relevance of HCV-RNA detection in PBMCs and/or the liver tissues in the absence of serum RNA positivity is currently poorly understood, the ability of the virus to replicate in these extrahepatic sites with the possible transmission risk to other tissues or other persons means that the awareness of OCI in clinical settings needs to be raised [34].

The significance of the persistence of HCV RNA in PBMCs in patients treated with DAAs who have achieved a SVR remains to be clarified. It is not clear if this finding may reflect an ongoing declining process of the virus inside tissues or whether it may represent a genuine permanent persistence of the virus inside the PBMCs with later relapse and/or the induction of complications. The finding of a very low virus level (less than 1000 u/ml) in PBMCs in all 20 patients with OCIs may favor the assumption of a declining process. However, the HCV RNA detected in the serum of one of our patients 6 months after SVR may support the other possibility, pointing to a real problem posed by the long-term persistence of the virus in this group of patients. This may necessitate a long-term follow-up study investigating the possible long-standing existence of HCV RNA in PBMCs in patients reported to have OCIs.

An important marker in HCV patients is the serum levels of kappa and lambda free light chains (FLC) of chronic HCV patients which means HCV infects B-lymphocytes, provokes cellular dysfunction and causes lymphoproliferative diseases. Persistence of viral antigenic stimulation in occult hepatitis c patient leads to worsening of progression of HCV infection to lymphoproliferative and/or autoimmune diseases [35].Detection of FLC in sera of OCI patients can inform us about the disease progression and induction of complication. FLC levels can be considered good biomarkers for identifying chronic HCV infection and its extrahepatic manifestation [36]. So we can compare the level of this marker between positive and negative HCV-RNA after they receive the DDA treatment in our future studies.

OCI remains a significant problem, even with highly effective DAAs, and it may play a major role in late relapse, cryptogenic hepatitis and other extrahepatic diseases. OCI in our study was significantly associated with elevated posttreatment serum transaminase levels. Liver cirrhosis is a significant risk factor for the occurrence of OCI following SVR. Ultimately, long-term studies to confirm the durability of an SVR after the use of interferon-free DAA regimens need to be conducted.

## Data Availability

The datasets generated during and/or analyzed in the current study are available from the corresponding author on reasonable request.
